# Personalized estimates of morphometric similarity in bipolar disorder and schizophrenia

**DOI:** 10.1038/s41537-020-00128-x

**Published:** 2020-12-04

**Authors:** Gaelle E. Doucet, Dongdong Lin, Yuhui Du, Zening Fu, David C. Glahn, Vincent D. Calhoun, Jessica Turner, Sophia Frangou

**Affiliations:** 1 Department of Psychiatry, Icahn School of Medicine at Mount Sinai, New York, NY USA; 2 Boys Town National Research Hospital, Omaha, NE USA; 3 Tri-Institutional Center for Translational Research in Neuroimaging and Data Science, Georgia State University, Georgia Institute of Technology, and Emory University, Atlanta, GA USA; 4 School of Computer & Information Technology, Shanxi University, Taiyuan, China; 5 Tommy Fuss Center for Neuropsychiatric Disease Research, Boston Children’s Hospital, Harvard University, Boston, MA USA; 6 Department of Psychology, Neuroscience Institute, Georgia State University, Atlanta, GA USA; 7 Centre for Brain Health, University of British Columbia, Vancouver, BC Canada

**Keywords:** Schizophrenia, Biomarkers, Psychosis

## Abstract

Bipolar disorder and schizophrenia are associated with brain morphometry alterations. This study investigates inter-individual variability in brain structural profiles, both within diagnostic groups and between patients and healthy individuals. Brain morphometric measures from three independent samples of patients with schizophrenia (*n* = 168), bipolar disorder (*n* = 122), and healthy individuals (*n* = 180) were modeled as single vectors to generated individualized profiles of subcortical volumes and regional cortical thickness. These profiles were then used to compute a person-based similarity index (PBSI) for subcortical volumes and for regional cortical thickness, to quantify the within-group similarity of the morphometric profile of each individual to that of the other participants in the same diagnostic group. There was no effect of diagnosis on the PBSI for subcortical volumes. In contrast, compared to healthy individuals, the PBSI for cortical thickness was lower in patients with schizophrenia (effect size = 0.4, *p* ≤ 0.0002), but not in patients with bipolar disorder. The results were robust and reproducible across samples. We conclude that disease mechanisms for these disorders produce modest inter-individual variations in brain morphometry that should be considered in future studies attempting to cluster patients in subgroups.

## Introduction

Bipolar disorder and schizophrenia are complex psychiatric disorders^1^ that rank among the leading causes of disease burden worldwide^2^. A substantial body of literature has established that both disorders are associated with brain structural alterations. These involve cortical thinning that is most pronounced in frontal and temporal regions and subcortical volume reductions, particularly in the thalamus and the hippocampus; the magnitude of these abnormalities is generally larger for schizophrenia than bipolar disorder^3–6^. However, case–control findings represent differences in group means which may not apply to each individual patient. The current emphasis on precision psychiatry^7,8^ has shifted the focus of analysis from groups to single individuals. Brain morphometry shows marked inter-individual variation in the general population that reflects the specific genetic and environmental background of each person^9^. Increased variance in regional morphometric measures, compared to healthy individuals, has been reported in schizophrenia involving primarily the cortical thickness of frontotemporal regions and the volume of the hippocampus and its subfields^10,11^. However, Wolfers and colleagues^12^, who examined individual-level deviation from normative gray matter volume values in patients with schizophrenia or bipolar disorder, found significant spatial convergence between individual- and group-level abnormalities. These studies treat the brain as a series of independent regions or voxels despite evidence of significant covariance between morphometric measures^13^. Machine learning algorithms attempt to address this limitation through the identification of multivariate brain structural patterns that might distinguish patients from healthy individuals^14^. Such studies have generally reported low accuracy and reproducibility^15–17^, especially with increasing sample size^18^, indicating that disease-related changes in multivariate neuroanatomical profiles are probably insufficient for reliable stratification.

The degree to which patients groups can be stratified using neuroanatomical measures deprends on the within-group similarity of their profiles. High levels of within-group morphometric similarity among patients would argue against significant heterogeneity at least in terms of neuroanatomical profiles. By contrast, low levels of within-group similarity among patients would be indicative of heterogeneity and would encourage attempts at stratification based on brain morphometry. Here we use a novel metric, the person-based similarity index (PBSI)^19^ to investigate the degree of within-group similarity (or otherwise) of an individual’s neuroanatomical profile. For each individual, their PBSI score quantifies the similarity between their brain structural profile and that of all other group members.

We have previously demonstrated that the PBSI based on brain morphometry metrics is biologically and functionally meaningful as it is reproducible and heritable^19^. Using this index, we examined the person-specific in-group similarity in regional cortical thickness (PBSI-CT) and subcortical volumes (PBSI-SV) in patients with schizophrenia (*n* = 93) or bipolar disorder (*n* = 44) and healthy individuals (*n* = 52) enrolled at the Icahn School of Medicine at Mount Sinai (ISMMS Discovery sample). Independently acquired data on schizophrenia (COBRE sample: patients = 75; health individuals = 87) and bipolar disorder (Yale sample: patients = 78; healthy individuals = 41) were used to test reproducibility (Table 1). We focused specifically on cortical thickness and subcortical volumes as these are the most widely used neuroimaging metrics for which there is robust evidence for diagnosis-related abnormalities in both bipolar disorder and schizophrenia^3–6^. Our assumption is that disease-related mechanisms interact with multiple processes that affect brain structure and that the outcome is likely to vary depending on each patient’s unique characteristics; accordingly, if disease mechanisms increase heterogeneity they should also increase the variance of brain morphometry in patients and reduce intra-group similarity in brain imaging profiles when compared to that expected in non-clinical samples.

**Table 1:** Site sample characteristics for each diagnostic group.

	ISMMS sample			Yale sample		COBRE sample	
	Healthy individuals *n* = 52	Patients with bipolar disorder *n* = 44	Patients with schizophrenia *n* = 93	Healthy individuals *n* = 41	Patients with bipolar disorder *n* = 78	Healthy individuals *n* = 87	Patients with schizophrenia *n* = 75
Male sex (*n*, %)	28 (53.8)	29 (65.9)	71 (76.3)	13 (31.7)	26 (33.3)	62 (71.3)	62 (82.7)
Age (mean, years)	29.8 (8.3)	27.6 (8.3)	27.3 (7.5)	33.2 (11.8)	34.2 (12.6)	38.2 (11.8)	37.9 (14.2)
IQ (mean)	115.2 (16.6)	103.0 (17.9)	93.4 (15.0)	104.1 (19.7)	106.0 (17.1)	111.0 (12.9)	97.9 (17.3)
BPRS total score (mean (std))	24.2 (0.4)	46.3 (19.8)	50.9 (19.6)	25.5 (3.9)	32.6 (8.7)	–	–
PANSS total score (mean (std))	–	–	–	–	–	–	59.7 (15.6)
Psychotic symptoms (*n*, %)	–	44 (100)	93 (100)	–	21 (26.9)	–	75 (100)
Unmedicated (*n*, %)	–	4 (9.3)	5 (5.4)	–	15 (19.2)	–	0 (0)
Lithium (*n*, %)	–	18 (41.9)	4 (3.9)	–	11 (14.1)	–	0 (0)
Antipsychotics (*n*, %)	–	34 (79.1)	78 (83.9)	–	24 (30.8)	–	66 (88.0)
Daily antipsychotic dose (mean, CPZE)	–	275.9 (339.3)	261.0 (205.4)	–	92.2 (224.5)	–	370.5 (296.3)

*BPRS* Brief Psychiatric Rating Scale, *COBRE* Center of Biomedical Research Excellence, *PANSS* Positive and Negative Syndrome Scale, *ISMMS* Icahn School of Medicine at Mount Sinai; patients were on more than one medication; additional details in Supplementary Tables S1–S3.

## Results

**Fig. 1:**
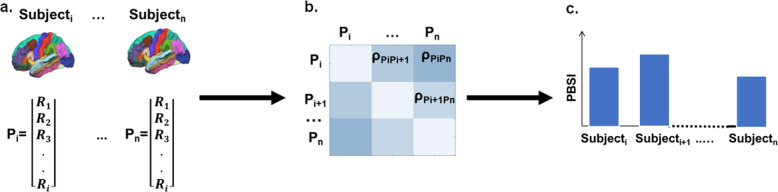
Pipeline for computing a person-based similarity index. The Person-Based Similarity Index (PBSI) quantifies the similarity of an individual’s morphometric profile to those of all other individuals in the same group. **a** Creation of a structural profile (P) using regional measures (R) (e.g., cortical thickness or subcortical volumes) for each individual *i*. **b** Computation of Spearman’s correlation ρ between each pair of individual profiles. **c** For each individual *i*, the person-based similarity index (PBSI) is computed as the average of all pairwise correlations between individual *i* and all other individuals within the same group.

MRI data from the ISMMS, the Yale, and COBRE samples were acquired on Siemens 3T scanners using similar protocols. The acquired data were processed separately using identical analysis protocols as described in the “Methods” section and in Supplementary Note 1. We did not use any harmonization method to remove site effects because we were interested in testing the replicability of findings across samples and show that the results were independent of acquisition protocol. Measures of cortical thickness and subcortical volume were extracted using the FreeSurfer v.5.3 image analysis suite. We followed a validated procedure as per Doucet et al.^19^. (see “Methods” section and Fig. 1) to derive a PBSI-CT and PBSI-SV score for each participant, which quantified the degree of the similarity of their individual cortical thickness and subcortical profiles to all other members of their diagnostic group. We assessed (a) the contribution of regional measures to the PBSI scores, (b) associations between PBSI scores with sex and age, and (c) the effect of diagnosis on the PBSI scores.

### Regional contributions to the PBSI

**Fig. 2:**
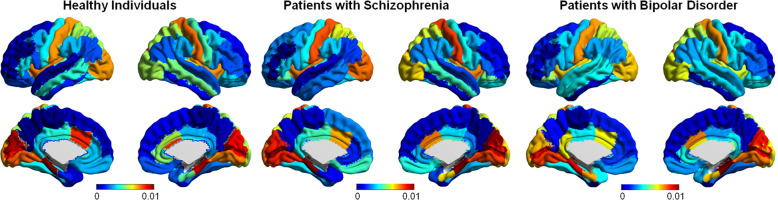
Contribution of regional cortical thickness measures to the person-based similarity index. In each participant within each diagnostic group, we used the leave-one-out approach to re-compute the person-based-similarity index for cortical thickness (PBSI-CT) after removing one regional cortical thickness measure at the time. We then calculated the absolute difference between each recalculated PBSI-CT and the original PBSI-CT (i.e., which included all cortical regions). The absolute mean of these difference scores in each diagnostic group is shown mapped on the cortical surface; warm colors reflect higher regional contributions. There were no significant differences in regional contributions between diagnostic groups.

We used a bootstrap resampling to examine whether the PBSI-CT and PBSI-SV scores were sensitive to the contribution of the regional morphometric measures. To do this, we created cortical thickness profiles for each individual by randomly selecting a subset of regional cortical thickness measures in increments of 10, from 10 to 60 regions. These analyses showed that no regional measure appeared to drive the PBSI-CT and PBSI-SV scores within each diagnostic group (Supplementary Fig. 1). Leave-one-out analyses revealed small (and not statistically significant), influences of regional measures; these were only present for cortical thickness and were independent of the diagnostic group (Supplementary Table 6 and Fig. 2). At the regional level, the coefficient of variation of the subcortical volume or cortical thickness measures did not significantly differ between the diagnostic groups (all *p*_FDR_ > 0.1). The PBSI-CT scores were positively correlated with variability in regional cortical thickness measures, but this effect was diagnosis-independent (*ρ* > 0.37, Supplementary Fig. 2).

### Association of PBSI scores with sex and age

The association between sex and age with PBSI scores was diagnosis-independent. An effect of age was observed only for the PBSI-CT in individuals 40 years or older regardless of diagnostic group. In this age group, the PBSI-CT was negatively associated with age in the ISMMS sample (Spearman *ρ* = −0.48, *p* = 0.03), the Yale sample (Spearman *ρ* = −0.20, *p* = 0.20) and the COBRE sample (Spearman *ρ* = −0.29, *p* = 0.01). Compared with men, women had higher PBSI-SV scores (Mann–Whitney *U* test, *Z* = 3.21, *p* = 2.4 × 10^−4^) and lower PBSI-CT scores (Mann–Whitney *U* test, *Z* = 6.1, *p* = 1.3 × 10^−^^9^) regardless of sample and diagnosis.

### Morphometric similarity between patients with bipolar disorder and healthy Individuals

**Fig. 3:**
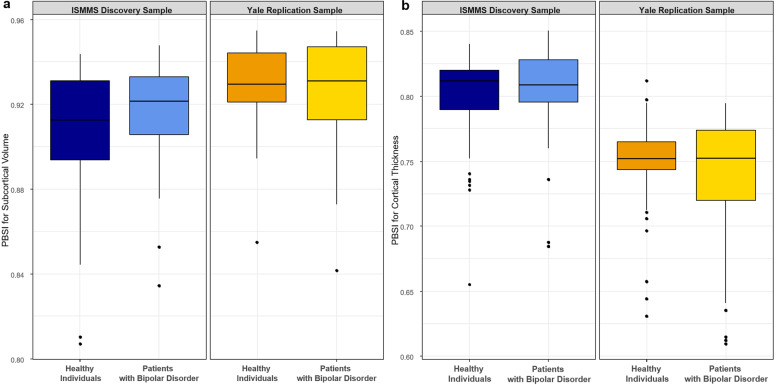
Person-based similarity index (PBSI) in bipolar disorder. **a** Subcortical volume; **b** cortical thickness. There were no significant case–control differences in either PBSI scores. The center line of the boxplot displays the median PBSI score, the bounds of the box show the 25th and 75th percentiles, the upper whisker is the maximum value of the data that is within 1.5 times the interquartile range over the 75th percentile. The lower whisker is the minimum value of the data that is within 1.5 times the interquartile range under the 25th percentile.

Patients with bipolar disorder and healthy individuals had comparable PBSI-CT and PBSI-SV both in the ISMMS discovery sample (Mann–Whitney *U* tests, PBSI-CT: *Z* = 0.7, *p*_unc_ = 0.5; PBSI-SV: *Z* = 1.1, *p*_unc_ = 0.2) and the Yale replication sample (Mann–Whitney *U* tests, PBSI-CT: *Z* = 0.03, *p*_unc_ = 0.9; PBSI-SV: *Z* = 0.07, *p*_unc_ = 0.9) (Fig. 3 and Supplementary Table 5, Supplementary Fig. 3). The exclusion of outliers did not alter the results.

In the ISMMS sample, all patients with bipolar disorder reported psychotic symptoms during mood episodes at some point during the course of their illness (Table 1). Consequently, the effect of psychosis was only investigated in the Yale sample (Table 1) in which patients with psychotic symptoms had higher PBSI-CT (Mann–Whitney *U* test, *Z* = 2.6, *p*_unc_ = 0.008) and PBSI-SV (Mann–Whitney *U* test, *Z* = 2.3, *p*_unc_ = 0.02) than those without psychotic symptoms, but these findings did not survive correction for multiple testing.

There was no difference in any of the PBSI scores between those patients who were prescribed lithium and those that were not in either sample (Mann–Whitney *U* tests, ISMMS: *p*_unc_ > 0.1; Yale: *p*_unc_ > 0.4l), even when not adjusting for multiple comparisons. Similarly, there was no association between any PBSI score and daily antipsychotic dose (ISMMS:|*ρ*| < 0.1, *p*_unc_ > 0.4; Yale: |*ρ*| < 0.2, Mann–Whitney *U* tests, all *p*_unc_ > 0.08).

There were no significant associations between any PBSI score and any Brief Psychiatric Rating Scale (BPRS) scores (total or subscale scores) (ISMMS: Spearman *ρ* range: −0.31, +0.17, *p*_FDR_ > 0.1; Yale: Spearman *ρ* range: −0.12, +0.13, *p*_FDR_ > 0.2).

### Morphometric similarity between patients with schizophrenia and healthy individuals

**Fig. 4:**
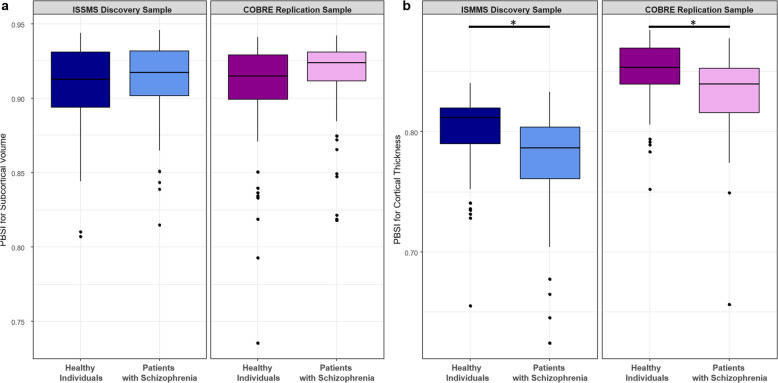
Person-based similarity index (PBSI) in schizophrenia. **a** Subcortical volume; **b** cortical thickness. *Significant case–control differences at *p*_FDR_ < 0.05. The center line of the boxplot displays the median PBSI score, the bounds of the box show the 25th and 75th percentiles, the upper whisker is the maximum value of the data that is within 1.5 times the interquartile range over the 75th percentile. The lower whisker is the minimum value of the data that is within 1.5 times the interquartile range under the 25th percentile.

The PBSI-SV scores from the patients with schizophrenia were comparable to those of healthy individuals both in the ISMMS discovery (Mann–Whitney *U* test, *Z* = 0.7, *p*_FDR_ = 0.5) and the COBRE replication (Mann–Whitney *U* test, *Z* = 2.0, *p*_FDR_ = 0.08) samples (Fig. 4 and Supplementary Table 5, Supplementary Fig. 3). By contrast, patients with schizophrenia had lower PBSI-CT scores than healthy individuals, in the ISMMS (Mann–Whitney *U* test, *Z* = −3.9, *p*_FDR_ = 0.0002; Cliff’s *d* = 0.40) and in the COBRE (Mann–Whitney *U* test, *Z* = −4, p_FDR_ = 10^−^^4^; Cliff’s *d* = 0.37) samples. This finding was robust to sex and showed no group by sex interaction (*p*_unc_ > 0.05); further, it was not driven by a specific region, based on the leave-one-out analyses, and was present in each lobe (Supplementary Table 7 and Supplementary Fig. 4). Exclusion of outliers did not alter the results.

In patients, neither PANSS scores nor antipsychotic dose were correlated with PBSI-CT or PBSI-SV in either sample (ISMMS sample: Spearman *ρ* range: 0.05, 0.21, *p*_FDR_ > 0.1; COBRE sample: Spearman *ρ* range: −0.17, 0.08, *p*_FDR_ > 0.2).

### Morphometric similarity between bipolar disorder and schizophrenia

We compared the PBSI scores between patients with schizophrenia and bipolar disorder in the ISMMS discovery sample. The PBSI-SV scores were comparable between the two diagnostic groups (Mann–Whitney *U* test, *Z* = −0.7; *p*_unc_ = 0.5). By contrast, patients with schizophrenia had lower PBSI-CT scores than patients with bipolar disorder (Mann–Whitney *U* test, *Z* = −4.5; *p*_unc_ = 7.10^−^^6^) (Supplementary Fig. 5).

## Discussion

We tested the within-group neuroanatomical similarity in patients with bipolar disorder, patients with schizophrenia, and healthy individuals. Within-group similarity was quantified at the level of person-based profiles of cortical thickness and subcortical volumes using a novel metric, the person-based similarity index (PBSI). The PBSI-CT and PBSI-SV, respectively, quantify the similarity of each individual’s cortical and subcortical profiles to those of all the other members of their diagnostic group. We demonstrated that these metrics were independent of regional variations in cortical thickness and volume. The PBSI-CT and PBSI-SV of patients with bipolar disorder were comparable to those of healthy individuals. Patients with schizophrenia had lower PBSI-CT, but no PBSI-SV, scores compared to healthy individuals. Importantly, these findings were reproducible across independent samples.

Both bipolar disorder and schizophrenia are considered heterogeneous disorders. Heterogeneity in bipolar disorder has been reported in genetic architecture^20,21^, cognitive profiles^22–26^, and clinical symptoms^27,28^. Likewise, heterogeneity in schizophrenia has been noted at the genetic^29^, cognitive^12,30^, and clinical level^31–33^. Several studies have linked variations in genetic, cognitive, and clinical features of patients with bipolar disorder^34–38^ or schizophrenia^38–43^ to a range of neuroanatomical features. These findings have been used to support the notion that patients differ fundamentally, rather than incrementally, from each other because of heterogeneity in the underlying etiological or pathophysiological mechanisms. There is emerging skepticism as to whether this is indeed the case. For example, a recent attempt to parse schizophrenia into subgroups defined by their neuroanatomy identified two subtypes of patients; these subtypes differed in the extent of the volumetric reductions along a continuum of severity and were associated with differences in IQ rather than any aspect of disease expression^44^. Additionally, studies that identified cognitive subtypes in either disorder have typically found that such subtypes were on a continuum of severity from non-impaired to having global deficits^30^. Importantly, disease-independent factors, such as age and education, seem to influence the nature and number of subgroups^30^.

In this study, we show that age and sex are important disease-indepedent sources of variability in neuroanatomical profiles. Replicating our prior findings in healthy individuals^19^, we show diagnosis-independent effects of sex and age on the inter-individual similarity in cortical thickness and subcortical volume profiles of patients suggesting that the within-group homogeneity of any sample is influenced by its demographic composition. It could therefore be argued that factors which are not related to pathogenesis are likely to drive much of the “heterogeneity” findings in bipolar disorder and in schizophrenia. The presence of variability in patient population is not sufficient to infer etiological heterogeneity as variations in disease presentation are present even in disorders with a single, clearly identifiable etiology, such as Huntington’s disease^45^ or tuberculosis^46^.

The findings of the present study also question whether there is indeed neuroanatomical heterogeneity in psychotic disorders. We focused on brain morphometry because structural MRI is widely used in research and clinical settings and has high translational potential. Unlike other studies, we generated person-based measures of within-group similarity, which enable individualized assessments of how similar (or otherwise) a patient might be compared to other members of the same diagnostic group. Patients with bipolar disorder showed within-group similarity, which was comparable to that of healthy individuals. There are two possible explanations for this. Bipolar disorder may be quite homogenous in terms of neuroanatomy, despite clinical variability. Alternatively, if etiological or pathophysiological heterogeneity in this disorder is present, it may not induce detectable within-group divergence in brain morphometry. In schizophrenia, within-group similarity, particularly for cortical thickness, was lower than that observed in healthy individuals. We, therefore, infer that disease-related mechanisms seem to increase divergence in cortical morphometry in this disorder. Although the effect size of case–control differences was small, it raises the possibility that there may be a minority of patients with schizophrenia that differ significantly from others with the same diagnosis. This possibility is supported by Janssen and colleagues^47^ who generated PBSI scores for cortical gyrification; most patients in their study had similar profiles to those of the healthy controls with the exception of a small subgroup that showed extreme deviance. Therefore, heterogeneity might be present in schizophrenia but may be limited to an “extreme” but a small subgroup that requires further study in larger samples.

The size of our samples was generally modest but the results were robust to replication suggesting that differences in the specific composition of the samples or MRI acquisition parameters did not have a major influence. There are multiple neuroimaging measures that could be examined for evidence of heterogeneity, which is not covered here. However, this study presents a methodological approach for future investigations of heterogeneity using a precision psychiatry approach that is not only applicable to neuroimaging but to other biological measures as well as at the voxel level. In sum, bipolar disorder showed minimal evidence of neuroanatomical heterogeneity in terms of patients’ global profiles. The neuroanatomical profiles of most patients with schizophrenia appeared largely similar to each other but hint at the possibility that a minority of patients may have different profiles. Their reliable identification would require very large samples.

## Method

### Samples

#### ISMMS discovery sample

The discovery sample was recruited at the Icahn School of Medicine at Mount Sinai (ISMMS), New York, USA. The sample comprised 93 patients with schizophrenia, 44 patients with psychotic bipolar disorder, Type I, and 52 healthy age- and sex-matched individuals (Table 1 and Supplementary Table 1). The diagnostic status of all participants according to the Diagnostic and Statistical Manual of Mental Disorders, Fifth Edition (DSM-5)^1^ was ascertained via personal interview using the Structured Clinical Interview for DSM-5^48^ supplemented by information from medical records in the case of patients. All participants were screened to exclude IQ < 70; the presence of a systemic medical illness or central nervous system disorder; a history of significant head trauma; DSM-5 substance use disorder and contra-indications for magnetic resonance imaging (MRI). In all participants, IQ was assessed using the Wechsler Abbreviated Scale of Intelligence^49^, and psychopathology was rated the 24-item BPRS^50^, which encompasses the entire range of psychopathology and is suitable for the assessment of non-clinical populations. Medication type and dose were recorded in patients and the daily antipsychotic dose was converted to chlorpromazine equivalents (CPZE)^51^. Further details on recruitment and assessment are provided in Supplementary Note 1.

#### Yale replication sample for bipolar disorder

The sample was recruited at the Olin Neuropsychiatric Research Center, Yale University, Hartford, CT, USA, and comprised 78 patients with bipolar disorder, Type I, and 41 healthy age- and sex-matched individuals (Table 1 and Supplementary Table 2). The diagnostic assessment and eligibility criteria in the Yale sample were identical to those used at the ISMMS.

#### COBRE replication sample for schizophrenia

A sample of 75 patients with schizophrenia and 87 healthy age- and sex-matched individuals (Table 1 and Supplementary Table 3) was provided by the Center of Biomedical Research Excellence (COBRE) (http://coins.trendscenter.org), which is an open-access collection of neuroimaging data in schizophrenia^52^. The diagnostic status of participants in the COBRE sample was ascertained according to the Diagnostic and Statistical Manual of Mental Disorders, Fourth Edition (DSM-IV)^53^ using the Structured Clinical Interview for DSM-IV Axis I Disorders^47^. All participants were screened to exclude those with a history of neurological disorder, mental retardation, severe head trauma, substance abuse, or dependence within the last 12 months and MRI contraindications. Psychopathology was assessed only in patients using the Positive and Negative Syndrome Scale (PANSS)^54^.

#### Ethics statement

The authors assert that all procedures contributing to this work comply with the ethical standards of the relevant national and institutional committees on human experimentation and with the Helsinki Declaration of 1975, as revised in 2008. At each site, the study was approved by the respective Institutional Review Board (ISMMS; Hartford Hospital and Yale University; University of New Mexico). All participants provided written informed consent prior to enrollment.

### Neuroimaging

The MRI data from each sample were acquired using Siemens 3T scanners (Erlangen, Germany) and were processed separately using identical analysis protocols as described in Supplementary Note 2. Cortical reconstruction based on the Desikan atlas^55^ and volumetric segmentation of structural data sets was implemented in the FreeSurfer image analysis suite (version 5.3.0; http://surfer.nmr.mgh.harvard.edu/). In each participant, 64 cortical thickness and 18 subcortical volume measures were extracted from the structural data set (detailed in Supplementary Table 4).

### Computation of the person-based similarity index (PBSI)

We followed a validated procedure as per Doucet et al.^19^ (Fig. 1). First, we concatenated cortical thickness and subcortical volume measures from each individual into two vectors to generate a person-specific profile of cortical thickness and subcortical volume. This procedure was independent of diagnosis or sample as it used only the individual’s data set. We treated regional cortical thickness and subcortical volume as separate phenotypes because current evidence suggests that they have partially distinct genetic, age-related, environmental, and clinical correlates^56–58^. The next steps were performed separately in each diagnostic group within each site sample because our intention was to evaluate within-group similarity in the cortical and subcortical profiles. For example, the profiles of the patients with bipolar disorder assessed at the ISMMS were analyzed together with those of the other patients with bipolar disorder from ISMMS. The same applied for healthy individuals and patients with schizophrenia, whose profiles were analyzed with those belonging to participants in the same diagnostic group within each site sample. Consequently, the cortical thickness profile and the subcortical volume profile of an individual were correlated with the respective profiles of all other individuals in the same diagnostic group within the same site sample using the Spearman’s correlation coefficient *ρ*. This process produced *n* − 1 correlation coefficients per individual and per profile, where *n* is the number of participants in the same diagnostic group within the same site sample. We then averaged the respective correlation coefficients to generate the PBSI score for cortical thickness (PBSI-CT) and the PBSI score for subcortical volumes (PBSI-SV) for each individual. These scores thus quantify the average similarity of the cortical and subcortical profiles of each individual to those of the other study participants in the same diagnostic group within the same sample site. The PBSI identifies relative interregional patterns, and is independent of global measures such as intracranial volume or mean cortical thickness. Higher scores denote greater similarity.

We used a bootstrap resampling to examine whether the PBSI-CT and PBSI-SV scores were sensitive to the contribution of the regional morphometric measures. To do this, we created cortical thickness profiles for each individual by randomly selecting a subset of regional cortical thickness measures in increments of 10, from 10 to 60 regions. For each diagnostic group within each site sample, we recalculated the PBSI-CT 100 times. Similarly, we created subcortical volume profiles for each individual by randomly grouping half of the variables (i.e., 8) and recalculated the PBSI-SV 100 times for each individual.

Further, in each diagnostic group within each site, we quantified the contribution of each morphometric measure to PBSI-CT and PBSI-SV by using the leave-one-out approach; this entailed recalculating the PBSI-CT and PBSI-SV scores for each individual after leaving out one regional brain measure at a time.

### Statistical analyses

All analyses were performed separately for the discovery and replication samples using identical procedures implemented in SPSS® v23.0 and in R. We employed the Kolmogorov–Smirnov test to evaluate data normalcy and implemented parametric (Student’s *t*-test) or non-parametric tests (Mann–Whitney *U* test), as appropriate, to identify group differences in continuous variables. An estimate of effect size for case–control differences was obtained using Cliff’s delta (*d*), which measures differences in the distribution of variable values between two samples^2^. Group differences in the distribution of categorical data were examined using chi-square tests.

The variability of each cortical thickness and subcortical volume measure in each diagnostic group within each sample was estimated by computing the coefficient of variation. Statistical differences in the regional coefficient of variation between diagnostic groups were evaluated using the asymptotic test for the equality of coefficient of variation^59^ (*cvequality* package in R-cran). Using either Spearman’s correlation analyses or Mann–Whitney *U* tests as appropriate, we assessed the association of PBSI scores with age, sex, and cortical thickness or subcortical volume measures in all participants and, in patients with symptom ratings, and medication status. Results were considered significant following the false-discovery rate (FDR) correction for multiple testing.

### Supplementary analyses

In the ISMMS sample only, we recalculated the PBSI scores (SV and CT, separately) for each patient, after combining both bipolar and schizophrenia groups. We then tested for group differences for each score. These results are reported in Supplementary Note 3 (Supplementary Fig. 6).

### Reporting summary

Further information on research design is available in the Nature Research Reporting Summary linked to this article.

## Supplementary information

Supplementary Material

Reporting Summary

## Data Availability

The data that support the findings of this study are available from the corresponding author upon reasonable request. The MATLAB function used to compute the PBSI score is available at https://www.mathworks.com/matlabcentral/fileexchange/69158-similarityscore. FreeSurfer image analysis suite (version 5.3.0; http://surfer.nmr.mgh.harvard.edu/) was used to extract the cortical thickness and subcortical volumes for each participant.
