# Supplementary material for: Personalized estimates of morphometric similarity in bipolar disorder and schizophrenia
Source: NPJ Schizophr. 2020 Dec 4;6:39. doi: 10.1038/s41537-020-00128-x (PMC7718905; doi:10.1038/s41537-020-00128-x)
Supplement: Supplementary file 1 — Supplementary Material [file 41537_2020_128_MOESM1_ESM.pdf]

Personalized Estimates of Morphometric Similarity in Bipolar Disorder and Schizophrenia

Supplementary Material

A. Supplementary Note 1

1. Samples

1.1. Discovery Sample: Icahn School of Medicine at Mount Sinai (ISMMS)

1.1.1 Recruitment and Assessment**:** One hundred eighty nine participants were recruited at the ISMMS, New York. The sample comprised 93 patients with schizophrenia, 44 patients with bipolar disorder, Type 1, and 52 healthy individuals (Supplementary Table 1). Patients were recruited via clinician referrals from the psychiatric services of the Mount Sinai Health System and healthy individuals were recruited by advertisement in the local press. The diagnostic status of all participants was based on diagnostic criteria outlined in the 5th edition of the Diagnostic and Statistical Manual of Mental Disorders (DSM-5), (1) following a personal interview with the Structured Clinical Interview for DSM-5, and (2) supplemented by information from medical records in the case of patients. The eligibility criteria for all participants were (a) 18-45 years; (b) able to communicate fluently in English; (c) IQ>70 based on the Wechsler Abbreviated Scale of Intelligence (3); (d) no history of head trauma or loss of consciousness; (e) no current or lifetime history of medical or neurological disorders; (f) no lifetime history of DSM-5 substance use disorder; (g) no MRI contra-indications (e.g. metal implants, claustrophobia). Healthy individuals were included if they had no lifetime personal history of mental disorders and no family history (up to second-degree relatives) of schizophrenia spectrum or any mood disorder. Medication type and dose were recorded in patients and the daily antipsychotic dose was converted to chlorpromazine equivalents (CPZE) (4). The BPRS items are coded as 1 (absent) to 7 (extremely severe). The BPRS subscale scores were the derived by summing up the scores of individual items as follows: BPRS positive symptoms=sum of scores for hallucination, unusual thought content, and bizarre behavior; BPRS negative symptoms=sum of scores for blunted affect, emotional withdrawal, and motor retardation; BPRS Depression/Anxiety Scores=sum of scores for anxiety, depression, suicidality, and guilt; BPRS Mania/Disorganization Scores=sum of scores for motor hyperactivity, elevated mood, excitement, distractibility, and grandiosity. The study was approved by the Institutional Review Board of the ISMMS; all participants provided written informed consent prior to study enrollment.

1.1.2 Neuroimaging Data Acquisition**:** For all participants, high-resolution structural images were acquired on a 3T Siemens Skyra scanner (Erlangen, Germany). Structural images were acquired using a T_1-weighted, 3D magnetization-prepared rapid gradient-echo (MPRAGE) sequence with the following parameters: Repetition time (TR)=2400ms; Time to Echo (TE)=2.07ms and Inversion time (TI)=1000ms, voxel size=0.8mm isotropic, flip angle=8°, field of view (FOV)=256×256×179mm^3, matrix size=320×320, bandwidth=240 Hz/Pixel, echo spacing=7.6ms, in-plane acceleration GRAPPA (GeneRalized Autocalibrating Partial Parallel Acquisition) factor 2.

Supplementary Table 1. Sample Characteristics- ISMMS Discovery Sample

	**Patients with Bipolar Disorder N=44**	**Patients with Schizophrenia N=93**	**Healthy Individuals N=52**
Age (years)	27.6 (8.3)	27.3 (7.5)	29.8 (8.5)
Sex (n, % male)	29 (65.9)	71 (76.3)	28.0 (58.3)
Full-Scale IQ	103.0 (17.9)	93.4 (15.0)	115.2 (16.6)
BPRS Total Score	46.3 (19.8)	50.9 (19.6)	24.2 (0.4)
BPRS Positive Symptoms Score	9.6 (5.7)	10.7 (5.5)	3.0 (0.0)
BPRS Negative Symptoms Score	4.0 (1.7)	6.8 (3.8)	3.0 (0.1)
BPRS Depression/Anxiety Score	8.7 (4.6)	9.0 (5.3)	4.0 (0.6)
BPRS Mania-Disorganization Score	11.9 (8.2)	8.0 (5.0)	5.0 (0.6)
Unmedicated (n,%)	4 (9.3)	5 (5.4)	n/a
Lithium (n,%)	18 (41.9)	4 (3.9)	n/a
Any Antidepressant (n,%)	10.0 (22.7)	12 (12.9)	n/a
Any Antipsychotic (n,%)	34 (79.1)	78 (83.9)	n/a
First Generation Antipsychotics (n,%)	10.0 (23.3)	30 (32.3)	n/a
Second Generation Antipsychotics (n,%)	31.0 (72.1)	74 (79.6)	n/a
Daily Antipsychotic Dose (in CPZE)	275.9 (339.3)	261.0 (205.4)	n/a

Continuous variables are shown as mean (standard deviation); BPRS=Brief Psychiatric Rating Scale; CPZE=chlorpromazine equivalents; patients were prescribed more than one medication

1.2. Yale Replication Sample for Bipolar Disorder

1.2.1. Sample Recruitment and Assessment**:** This sample comprised 81 patients with bipolar disorder Type I disorder and 44 healthy individuals (Supplementary Table 2). Patients were recruited via clinician referrals from psychiatric facilities at Hartford Hospital, Connecticut. Healthy individuals were recruited from the local community via advertisements. The eligibility criteria applied were the same as for the discovery sample. The study was approved by the Institutional Review Board at Hartford Hospital and Yale University. All participants provided written informed consent.

1.2.2. Neuroimaging Data Acquisition**:** For all participants, high-resolution structural images were acquired on a 3T Siemens Alegra scanner (Erlangen, Germany). Structural images were acquired using a T_1-weighted, 3D MPRAGE sequence with the following parameters: TR/TE/TI= 2200/4.13/766ms, voxel size=0.8mm isotropic, flip angle=13°, image size=240×320×208 voxels.

**Supplementary Table 2: Sample Characteristics-Yale Replication Sample for Bipolar Disorder**		
	**Patients with Bipolar Disorder N=78**	**Healthy Individuals N=41**
Age (years)	34.2 (12.6)	33.2 (11.8)
Sex (n, % male)	26.0 (33.3)	13.0 (31.7)
Full-Scale IQ	106.0 (17.1)	104.1 (19.7)
BPRS Total Score	32.6 (8.7)	25.5 (3.9)
BPRS Positive Symptoms Score	3.2 (0.6)	3.0 (0.0)
BPRS Negative Symptoms Score	3.4 (0.9)	3.2 (0.6)
BPRS Depression/Anxiety Score	7.1 (3.5)	4.2 (0.5)
BPRS Mania-Disorganization Score	6.9 (3.4)	5.2 (0.7)
Unmedicated (n,%)	15.0 (19.2)	n/a
Lithium (n,%)	11.0 (14.1)	n/a
Any Antidepressant (n,%)	22.0 (27.1)	n/a
Any Antipsychotic (n,%)	24.0 (30.8)	n/a
First Generation Antipsychotics (n,%)	0.0 (0.0)	n/a
Second Generation Antipsychotics (n,%)	24.0 (30.8)	n/a
Daily Antipsychotic Dose (in CPZE)	92.2 (224.5)	n/a
Continuous variables are shown as mean (standard deviation); BPRS=Brief Psychiatric Rating Scale; CPZE=chlorpromazine equivalents; patients were prescribed more than one medication		

1. 3. COBRE Replication Sample for Schizophrenia

1.3.1 Sample Recruitment and Assessment**:** A sample of 76 patients with schizophrenia and 87 healthy individuals was provided by the Center of Biomedical Research Excellence (COBRE) (http://cobre.mrn.org; http://coins.mrn.org), which is an open-access collection of neuroimaging data in schizophrenia (5) (Supplementary Table 3). The diagnostic status of the COBRE participants was ascertained according to the Diagnostic and Statistical Manual of Mental Disorders, Fourth Edition (DSM-IV; APA 2000) using the Structured Clinical Interview using the Structured Clinical Interview for DSM-IV Axis I Disorders (6). Healthy individuals did not have any personal or family (up to second degree relatives) of psychiatric disorders. All participants were screened to exclude those with a history of neurological disorder, mental retardation, severe head trauma, substance abuse or dependence within the last 12 months and MRI contra-indications. Psychopathology in patients assessed with the Positive and Negative

Syndrome Scale (PANSS) (7). The COBRE Stability Clinic determined retrospective stability from relevant psychiatric records documenting that no change in symptomatology or type/dose of psychotropic medications occurred during the three months prior to the referral. IQ was assessed with Wechsler Abbreviated Scale of Intelligence (3). The study was approved by the Institutional Review Board of the University of New Mexico. All participants provided written informed consent prior to participating.

1.3.2. Neuroimaging Data Acquisition**:** All participants were scanned at the Mind Research Network using a 3T Siemens TIM Trio scanner. The structural data were acquired using a multi-echo (number of echos=5) MPRAGE (MEMPR) sequence with the following parameters: TR=2530ms; five TE=1.64, 3.5, 5.36, 7.22, 9.08ms, TI=900ms, 1mm isotropic resolution. FOV=256x256, flip angle=7°, matrix size=256x256x176, pixel bandwidth=650 Hz. With 5 echoes, the TR, TI and time to encode partitions for the MEMPR are similar to that of a conventional MPRAGE, resulting in similar contrast in the gray and white matter and cerebrospinal fluid.

Supplementary Table 3. Sample Characteristics- COBRE Replication Sample for Schizophrenia

	**Patients with Schizophrenia N=75**	**Healthy Individuals N=87**
Age (years)	37.9 (14.2)	38.2 (11.8)
Sex (n, % male)	62 (82.7)	62 (71.3)
Full-Scale IQ	97.9 (17.3)	111.0 (12.9)
PANSS Total Score	59.7 (15.6)	-
PANSS Positive Symptoms Score	15.3 (4.7)	-
PANSS Negative Symptoms Score	15.1 (5.4)	-
PANSS General Symptoms Score	59.7 (15.6)	-
Any Antidepressant (n,%)	n.a	-
Any Antipsychotic (n,%)	n.a	-
First Generation Antipsychotics (n,%)	n.a	-
Second Generation Antipsychotics (n,%)	n.a	-
Daily Antipsychotic Dose (in CPZE)	370.5 (296.3)	-

Supplementary Table 3. Sample Characteristics- COBRE Replication Sample for Schizophrenia		
	**Patients with Schizophrenia N=75**	**Healthy Individuals N=87**
Continuous variables are shown as mean (standard deviation); PANSS=Positive and Negative Syndrome Scale; CPZE=chlorpromazine equivalents; patients were prescribed more than one medication		

B. Supplementary Note 2

MRI Segmentation and Quality Assurance

Neuroimaging data from each sample were processed separately using an identical protocol implemented in FreeSurfer 5.3.0 (http://surfer.nmr.mgh.harvard.edu/). The steps included removal of non-brain tissue using a hybrid watershed/surface deformation procedure (8), automated Talairach transformation, segmentation of the subcortical white matter and deep gray matter volumetric structures (9, 10) intensity normalization (11), tessellation of the boundary between the gray and white matter, automated topology correction (12, 13), and surface deformation following intensity gradients to optimally place the gray/white matter boundaries and gray/cerebrospinal fluid borders at the location where the greatest shift in intensity defines the transition to the other tissue class. This process yielded 64 cortical thickness measures per hemisphere and 18 subcortical volume measures (Supplementary Table 4). Quality control for each structural dataset was implemented according to the publically available protocol from the ENIGMA initiative (http://enigma.ini.usc.edu/).

As the PBSI identifies relative interregional patterns, global measures such as total intracranial volume (ICV) or thickness have no effect on the PBSI. This was also empirically confirmed by repeating the analyses with and without adjustment for global measures. For subcortical volumes were adjusted for variation in intracranial volume (ICV) in accordance to Pintzka et al. (14) using the following equation: $Vol_{adj} = Vol - \beta * (ICV - \overline{ICV})$, where Vol_{adj} is the ICV-adjusted volume, *Vol* is the original uncorrected volume, β is the slope from the linear regression of Vol on ICV, ICV is the ICV of a study participant and $\overline{ICV}$ is the mean ICV across all study participants. Adjustment using whole brain gray matter volume (instead of ICV) yielded the same results and therefore is not reported. Adjustment for regional cortical thickness followed the same formula substituting ICV for mean cortical thickness.

Supplementary Table 4. Definition of the imaging variables	
**Cortical Thickness**	
Caudal anterior cingulate cortex	Derived from cortical reconstruction using Freesurfer v5.3.; left and right measures were considered separately
Caudal middle frontal gyrus	Derived from cortical reconstruction using Freesurfer v5.3.; left and right measures were considered separately
Cuneus	Derived from cortical reconstruction using Freesurfer v5.3.; left and right measures were considered separately
Entorhinal cortex	Derived from cortical reconstruction using Freesurfer v5.3.; left and right measures were considered separately
Fusiform gyrus	Derived from cortical reconstruction using Freesurfer v5.3.; left and right measures were considered separately
Inferior parietal lobule	Derived from cortical reconstruction using Freesurfer v5.3.; left and right measures were considered separately
Inferior temporal gyrus	Derived from cortical reconstruction using Freesurfer v5.3.; left and right measures were considered separately
Lateral occipital gyrus	Derived from cortical reconstruction using Freesurfer v5.3.; left and right measures were considered separately
Lateral orbitofrontal gyrus	Derived from cortical reconstruction using Freesurfer v5.3.; left and right measures were considered separately
Lingual gyrus	Derived from cortical reconstruction using Freesurfer v5.3.; left and right measures were considered separately
Medial orbitofrontal gyrus	Derived from cortical reconstruction using Freesurfer v5.3.; left and right measures were considered separately
Middle temporal gyrus	Derived from cortical reconstruction using Freesurfer v5.3.; left and right measures were considered separately
Parahippocampal gyrus	Derived from cortical reconstruction using Freesurfer v5.3.; left and right measures were considered separately
Paracentral gyrus	Derived from cortical reconstruction using Freesurfer v5.3.; left and right measures were considered separately
Pars opercularis	Derived from cortical reconstruction using Freesurfer v5.3.; left and right measures were considered separately
Pars orbitalis	Derived from cortical reconstruction using Freesurfer v5.3.; left and right measures were considered separately
Pars triangularis	Derived from cortical reconstruction using Freesurfer v5.3.; left and right measures were considered separately

**Supplementary Table 4. Definition of the imaging variables**	
Pericalcarine gyrus	Derived from cortical reconstruction using Freesurfer v5.3.; left and right measures were considered separately
Postcentral gyrus	Derived from cortical reconstruction using Freesurfer v5.3.; left and right measures were considered separately
Posterior cingulate cortex	Derived from cortical reconstruction using Freesurfer v5.3.; left and right measures were considered separately
Precentral gyrus	Derived from cortical reconstruction using Freesurfer v5.3.; left and right measures were considered separately
Precuneus	Derived from cortical reconstruction using Freesurfer v5.3.; left and right measures were considered separately
Rostral anterior cingulate cortex	Derived from cortical reconstruction using Freesurfer v5.3.; left and right measures were considered separately
Rostral middle frontal gyrus	Derived from cortical reconstruction using Freesurfer v5.3.; left and right measures were considered separately
Superior frontal gyrus	Derived from cortical reconstruction using Freesurfer v5.3.; left and right measures were considered separately
Superior parietal lobule	Derived from cortical reconstruction using Freesurfer v5.3.; left and right measures were considered separately
Superior temporal gyrus	Derived from cortical reconstruction using Freesurfer v5.3.; left and right measures were considered separately
Supramarginal gyrus	Derived from cortical reconstruction using Freesurfer v5.3.; left and right measures were considered separately
Frontal pole	Derived from cortical reconstruction using Freesurfer v5.3.; left and right measures were considered separately
Temporal pole	Derived from cortical reconstruction using Freesurfer v5.3.; left and right measures were considered separately
Transverse temporal gyrus	Derived from cortical reconstruction using Freesurfer v5.3.; left and right measures were considered separately
Insula	Derived from cortical reconstruction using Freesurfer v5.3.; left and right measures were considered separately
**Subcortical Volumes**	
Thalamus	Derived from segmentation using Freesurfer v5.3.; left and right measures were considered separately following ICV correction
Hippocampus	Derived from segmentation using Freesurfer v5.3.; left and right measures were considered separately following ICV correction

**Supplementary Table 4. Definition of the imaging variables**	
Caudate nucleus	Derived from segmentation using Freesurfer v5.3.; left and right measures were considered separately following ICV correction
Nucleus Accumbens	Derived from segmentation using Freesurfer v5.3.; left and right measures were considered separately following ICV correction
Pallidum	Derived from segmentation using Freesurfer v5.3.; left and right measures were considered separately following ICV correction
Putamen	Derived from segmentation using Freesurfer v5.3.; left and right measures were considered separately following ICV correction
Amygdala	Derived from segmentation using Freesurfer v5.3.; left and right measures were considered separately following ICV correction
Lateral ventricles	Derived from segmentation using Freesurfer v5.3.; left and right measures were considered separately following ICV correction

C. Supplementary Note 3: Supplementary Results

1. Descriptive Statistics and Stability of the Person-Based Similarity Index (PBSI)

The descriptive statistics for the PBSI for subcortical volume (PBSI-SV) and cortical thickness (PBSI-CT) are shown in Supplementary Table 5.

Supplementary Table 5. Person-Based Similarity Index (PBSI) Scores for Each Diagnostic Group and Each Site Sample

Sample	PBSI for Subcortical Volume	PBSI for Cortical Thickness
**ISMMS Discovery Sample**		
Patients with Bipolar Disorder	Mean (sd)=0.916 (0.024)<br>Median=0.922	Mean (sd)=0.808 (0.036)<br>Median=0.809
Patients with Schizophrenia	Mean (sd)=0.913 (0.026)<br>Median=0.919	Mean (sd) =0.778 (0.039)<br>Median=0.787
Healthy Individuals	Mean (sd)=0.910 (0.028)<br>Median=0.918	Mean (sd)=0.798 (0.035)<br>Median=0.812
**Yale Replication Sample for Bipolar Disorder**		
Healthy Individuals	Mean (sd) =0.929 (0.019)<br>Median=0.929	Mean (sd)=0.733 ().072)<br>Median=0.751
Patients with Bipolar Disorder	Mean (sd)=0.927 (0.024)<br>Median=0.931	Mean (sd)=0.735 (0.053)<br>Median=0.752
**COBRE Replication Sample for Schizophrenia**		
Healthy Individuals	Mean (sd)=0.900 (0.052)<br>Median=0.914	Mean (sd)=0.850 (0.025)<br>Median=0.854
Patients with Schizophrenia	Mean (sd)=0.913 (0.031)<br>Median=0.924	Mean (sd) =0.832 (0.330)<br>Median=0.840

COBRE= Center of Biomedical Research Excellence; ISMMS=Icahn School of Medicine at Mount Sinai; SD=Standard Deviation

2. Contribution of Regional Morphometric Measures to the PBSI

To examine whether the PBSI-CT and PBSI-SV scores were sensitive to the contribution of each regional morphometric measure, we used a bootstrap resampling approach. We created cortical thickness profiles for each individual by randomly grouping regional cortical thickness measures in increments of 10, from 10 to 60 regions. We then recalculated the PBSI-CT 100 times. Similarly, we created subcortical volume profiles for each individual by randomly grouping half of the variables (i.e., 8) and recalculated the PBSI-SV 100 times for each individual. The results are shown in Supplementary Figure 1. The results shown are from the ISMMS discovery sample and were similar in the Yale and COBRE replication samples. Further, we used the leave-one-out approach to establish the contribution of each morphometric measure to their corresponding PBSI score in the ISMMS sample. The results are shown in Supplementary Table 6 and Figure 2 in the main text. Finally, we used the coefficient of variation to quantify variability in regional cortical thickness and subcortical volume in each diagnostic group. We found that regional contributions to the PBSI-CT were correlated with regional cortical thickness;

these associations were comparable across the three diagnostic groups in the ISMMS sample (Supplementary Figure 2).

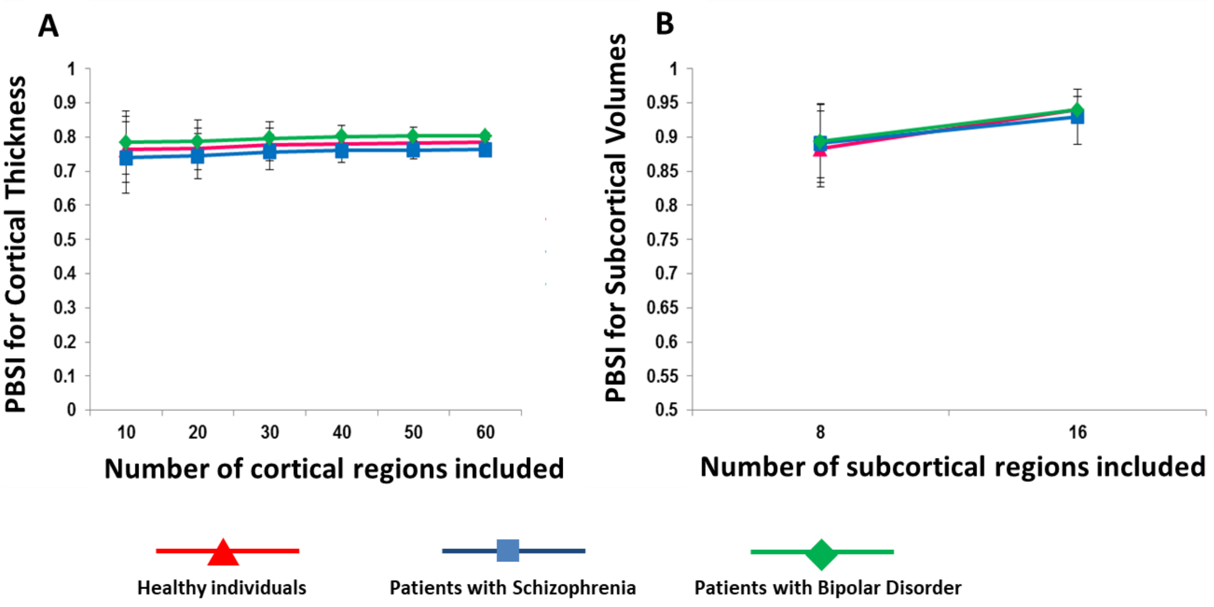

Supplementary Figure 1: Stability of person-based similarity index (PBSI) scores to the contribution of regional morphometric measures. (A) Recomputed PBSI for cortical thickness based on cortical thickness profiles generated by resampling cortical thickness measures; (B) Recomputed PBSI for subcortical volumes based on subcortical volume profiles generated by resampling subcortical volumes. Error bars represent standard deviation of the PBSI scores within each diagnostic group computed on 100 resampled datasets for each individual group member. Based on the ISMMS Discovery Samples.

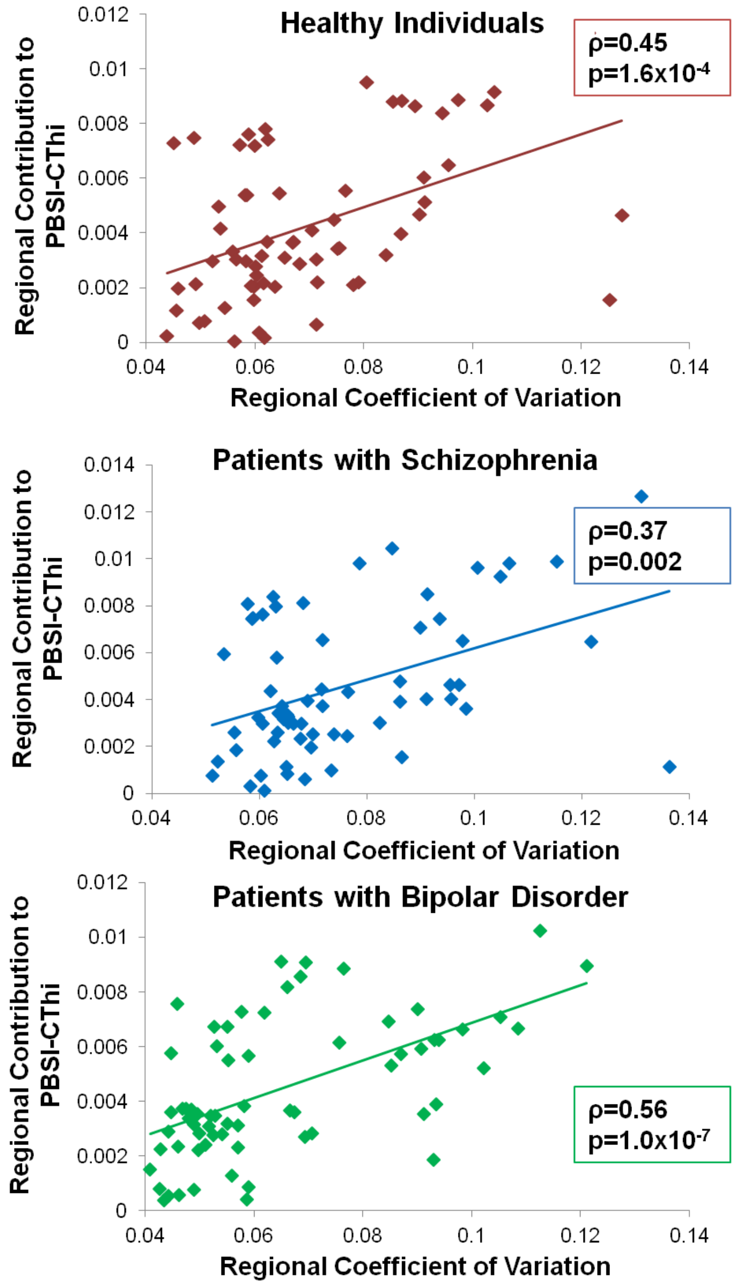

Supplementary Figure 2: Association between regional coefficient of variation in cortical thickness and regional contribution to the person-based similarity score for cortical thickness (PBSI-CT).

Regional coefficient of variation in each diagnostic group in the discovery ISMMS sample was calculated as the ratio of standard deviation to mean cortical thickness in each region. Spearman's correlation coefficient between this measure and regional contribution to the PBSI-CT were significantly correlated. The pattern was similar across diagnostic groups.

Supplementary Table 6: Regional Contributions to the Person Based Similarity Index (PBSI)						
	**Healthy Individuals**		**Schizophrenia**		**Bipolar Disorder**	
**Variable removed in the leave-one-out analyses**	**Recalculated PBSI following removal of the region shown in column 1**	**Regional Contribution to the PBSI Score***	**Recalculated PBSI following removal of the region shown in column 1**	**Regional Contribution to the PBSI Score ***	**Recalculated PBSI following removal of the region shown in column 1**	**Regional Contribution to the PBSI Score ***
**lh_parahippocampal_thickness**	0.798(0.058)	0.011(0.058)	0.78(0.073)	0.013(0.073)	0.813(0.036)	0.009(0.036)
**lh_cuneus_thickness**	0.777(0.06)	0.01(0.06)	0.758(0.071)	0.008(0.071)	0.797(0.035)	0.007(0.035)
**rh_parahippocampal_thickness**	0.796(0.057)	0.009(0.057)	0.777(0.073)	0.01(0.073)	0.815(0.037)	0.01(0.037)
**lh_pericalcarine_thickness**	0.778(0.059)	0.009(0.059)	0.756(0.076)	0.01(0.076)	0.795(0.038)	0.009(0.038)
**rh_pericalcarine_thickness**	0.778(0.059)	0.009(0.059)	0.758(0.074)	0.009(0.074)	0.795(0.038)	0.009(0.038)
**rh_cuneus_thickness**	0.778(0.06)	0.009(0.06)	0.757(0.076)	0.01(0.076)	0.796(0.037)	0.009(0.037)
**rh_temporalpole_thickness**	0.778(0.059)	0.009(0.059)	0.757(0.076)	0.01(0.076)	0.798(0.034)	0.007(0.034)
**lh_temporalpole_thickness**	0.778(0.059)	0.009(0.059)	0.757(0.076)	0.01(0.076)	0.796(0.037)	0.009(0.037)
**lh_caudalanteriorcingulate_thickness**	0.795(0.057)	0.008(0.057)	0.774(0.073)	0.007(0.073)	0.811(0.036)	0.006(0.036)
**lh_lingual_thickness**	0.779(0.059)	0.008(0.059)	0.758(0.073)	0.008(0.073)	0.796(0.037)	0.008(0.037)
**rh_postcentral_thickness**	0.779(0.06)	0.008(0.06)	0.759(0.075)	0.008(0.075)	0.797(0.037)	0.007(0.037)
**lh_insula_thickness**	0.78(0.06)	0.007(0.06)	0.759(0.075)	0.007(0.075)	0.798(0.037)	0.007(0.037)
**rh_lingual_thickness**	0.78(0.058)	0.007(0.058)	0.759(0.073)	0.008(0.073)	0.797(0.037)	0.008(0.037)
**rh_insula_thickness**	0.78(0.06)	0.007(0.06)	0.759(0.075)	0.007(0.075)	0.799(0.037)	0.006(0.037)
**lh_postcentral_thickness**	0.78(0.059)	0.007(0.059)	0.759(0.075)	0.008(0.075)	0.797(0.037)	0.007(0.037)
**lh_lateraloccipital_thickness**	0.78(0.06)	0.007(0.06)	0.759(0.075)	0.008(0.075)	0.798(0.037)	0.007(0.037)
**rh_transversetemporal_thickness**	0.793(0.057)	0.006(0.057)	0.772(0.074)	0.005(0.074)	0.811(0.036)	0.006(0.036)
**rh_frontalpole_thickness**	0.793(0.058)	0.006(0.058)	0.773(0.073)	0.007(0.073)	0.81(0.036)	0.006(0.036)
**rh_caudalanteriorcingulate_thickness**	0.793(0.058)	0.006(0.058)	0.774(0.072)	0.007(0.072)	0.812(0.035)	0.007(0.035)
**rh_superiorparietal_thickness**	0.782(0.059)	0.005(0.059)	0.761(0.075)	0.006(0.075)	0.799(0.037)	0.006(0.037)
**rh_lateraloccipital_thickness**	0.782(0.059)	0.005(0.059)	0.76(0.075)	0.007(0.075)	0.798(0.037)	0.006(0.037)

Supplementary Table 6: Regional Contributions to the Person Based Similarity Index (PBSI)						
	**Healthy Individuals**		**Schizophrenia**		**Bipolar Disorder**	
**Variable removed in the leave-one-out analyses**	**Recalculated PBSI following removal of the region shown in column 1**	**Regional Contribution to the PBSI Score***	**Recalculated PBSI following removal of the region shown in column 1**	**Regional Contribution to the PBSI Score ***	**Recalculated PBSI following removal of the region shown in column 1**	**Regional Contribution to the PBSI Score ***
**lh_superiorparietal_thickness**	0.782(0.059)	0.005(0.059)	0.761(0.075)	0.006(0.075)	0.799(0.037)	0.006(0.037)
**rh_rostralanteriorcingulate_thickness**	0.792(0.058)	0.005(0.058)	0.77(0.074)	0.003(0.074)	0.808(0.036)	0.004(0.036)
**rh_middletemporal_thickness**	0.782(0.059)	0.005(0.059)	0.763(0.074)	0.004(0.074)	0.801(0.036)	0.003(0.036)
**lh_transversetemporal_thickness**	0.792(0.058)	0.005(0.058)	0.771(0.074)	0.004(0.074)	0.81(0.036)	0.006(0.036)
**rh_entorhinal_thickness**	0.782(0.056)	0.005(0.056)	0.76(0.07)	0.006(0.07)	0.798(0.036)	0.007(0.036)
**rh_precentral_thickness**	0.791(0.057)	0.004(0.057)	0.771(0.073)	0.004(0.073)	0.808(0.035)	0.004(0.035)
**lh_middletemporal_thickness**	0.783(0.059)	0.004(0.059)	0.764(0.072)	0.002(0.072)	0.801(0.036)	0.004(0.036)
**lh_posteriorcingulate_thickness**	0.791(0.058)	0.004(0.058)	0.771(0.072)	0.004(0.072)	0.808(0.035)	0.004(0.035)
**lh_frontalpole_thickness**	0.791(0.059)	0.004(0.059)	0.771(0.074)	0.004(0.074)	0.81(0.036)	0.005(0.036)
**lh_precentral_thickness**	0.791(0.057)	0.004(0.057)	0.771(0.073)	0.004(0.073)	0.808(0.035)	0.003(0.035)
**lh_fusiform_thickness**	0.791(0.057)	0.004(0.057)	0.77(0.073)	0.003(0.073)	0.808(0.035)	0.004(0.035)
**lh_parstriangularis_thickness**	0.791(0.056)	0.004(0.056)	0.77(0.073)	0.003(0.073)	0.807(0.036)	0.002(0.036)
**lh_isthmuscingulate_thickness**	0.79(0.057)	0.003(0.057)	0.771(0.073)	0.004(0.073)	0.811(0.034)	0.006(0.034)
**rh_parsorbitalis_thickness**	0.79(0.058)	0.003(0.058)	0.772(0.073)	0.005(0.073)	0.808(0.035)	0.004(0.035)
**rh_caudalmiddlefrontal_thickness**	0.79(0.057)	0.003(0.057)	0.77(0.074)	0.003(0.074)	0.808(0.035)	0.004(0.035)
**lh_rostralanteriorcingulate_thickness**	0.79(0.059)	0.003(0.059)	0.768(0.073)	0.002(0.073)	0.806(0.037)	0.002(0.037)
**lh_supramarginal_thickness**	0.79(0.057)	0.003(0.057)	0.77(0.073)	0.003(0.073)	0.807(0.035)	0.003(0.035)
**rh_lateralorbitofrontal_thickness**	0.79(0.058)	0.003(0.058)	0.771(0.073)	0.004(0.073)	0.808(0.035)	0.004(0.035)
**rh_posteriorcingulate_thickness**	0.79(0.057)	0.003(0.057)	0.77(0.073)	0.003(0.073)	0.807(0.035)	0.003(0.035)
**lh_medialorbitofrontal_thickness**	0.79(0.056)	0.003(0.056)	0.771(0.071)	0.004(0.071)	0.807(0.035)	0.003(0.035)
**rh_supramarginal_thickness**	0.79(0.057)	0.003(0.057)	0.77(0.073)	0.003(0.073)	0.808(0.036)	0.003(0.036)
**lh_caudalmiddlefrontal_thickness**	0.79(0.057)	0.003(0.057)	0.77(0.074)	0.003(0.074)	0.808(0.035)	0.003(0.035)

Supplementary Table 6: Regional Contributions to the Person Based Similarity Index (PBSI)						
	Healthy Individuals		Schizophrenia		Bipolar Disorder	
**Variable removed in the leave-one-out analyses**	**Recalculated PBSI following removal of the region shown in column 1**	**Regional Contribution to the PBSI Score***	**Recalculated PBSI following removal of the region shown in column 1**	**Regional Contribution to the PBSI Score ***	**Recalculated PBSI following removal of the region shown in column 1**	**Regional Contribution to the PBSI Score ***
**rh_isthmuscingulate_thickness**	0.79(0.056)	0.003(0.056)	0.769(0.073)	0.003(0.073)	0.808(0.035)	0.004(0.035)
**rh_parsopercularis_thickness**	0.79(0.058)	0.003(0.058)	0.77(0.073)	0.003(0.073)	0.808(0.036)	0.003(0.036)
**rh_fusiform_thickness**	0.789(0.057)	0.002(0.057)	0.77(0.073)	0.003(0.073)	0.807(0.035)	0.002(0.035)
**rh_superiortemporal_thickness**	0.785(0.056)	0.002(0.056)	0.765(0.073)	0.002(0.073)	0.801(0.036)	0.003(0.036)
**lh_parsorbitalis_thickness**	0.789(0.058)	0.002(0.058)	0.772(0.072)	0.005(0.072)	0.81(0.034)	0.005(0.034)
**rh_inferiorparietal_thickness**	0.789(0.057)	0.002(0.057)	0.77(0.073)	0.003(0.073)	0.807(0.036)	0.003(0.036)
**rh_rostralmiddlefrontal_thickness**	0.785(0.058)	0.002(0.058)	0.766(0.072)	0.001(0.072)	0.802(0.035)	0.002(0.035)
**rh_medialorbitofrontal_thickness**	0.789(0.055)	0.002(0.055)	0.771(0.07)	0.004(0.07)	0.807(0.034)	0.003(0.034)
**rh_parstriangularis_thickness**	0.789(0.056)	0.002(0.056)	0.769(0.073)	0.002(0.073)	0.807(0.036)	0.002(0.036)
**lh_inferiorparietal_thickness**	0.789(0.057)	0.002(0.057)	0.769(0.073)	0.002(0.073)	0.807(0.036)	0.003(0.036)
**lh_lateralorbitofrontal_thickness**	0.789(0.058)	0.002(0.058)	0.769(0.073)	0.003(0.073)	0.807(0.035)	0.002(0.035)
**lh_parsopercularis_thickness**	0.789(0.058)	0.002(0.058)	0.77(0.073)	0.003(0.073)	0.808(0.036)	0.003(0.036)
**lh_inferiortemporal_thickness**	0.789(0.058)	0.002(0.058)	0.769(0.073)	0.002(0.073)	0.804(0.036)	0.001(0.036)
**lh_entorhinal_thickness**	0.785(0.056)	0.002(0.056)	0.766(0.069)	0.001(0.069)	0.797(0.037)	0.007(0.037)
**rh_inferiortemporal_thickness**	0.786(0.058)	0.001(0.058)	0.767(0.072)	0.001(0.072)	0.804(0.036)	0(0.036)
**rh_superiorfrontal_thickness**	0.788(0.058)	0.001(0.058)	0.766(0.075)	0.001(0.075)	0.805(0.036)	0.001(0.036)
**rh_precuneus_thickness**	0.786(0.058)	0.001(0.058)	0.767(0.073)	0(0.073)	0.804(0.036)	0.001(0.036)
**lh_superiorfrontal_thickness**	0.786(0.059)	0.001(0.059)	0.764(0.075)	0.003(0.075)	0.803(0.037)	0.002(0.037)
**lh_superiortemporal_thickness**	0.786(0.054)	0.001(0.054)	0.766(0.072)	0.001(0.072)	0.801(0.037)	0.004(0.037)
**rh_paracentral_thickness**	0.787(0.057)	0(0.057)	0.768(0.073)	0.001(0.073)	0.805(0.037)	0.001(0.037)
**lh_rostralmiddlefrontal_thickness**	0.787(0.057)	0(0.057)	0.767(0.073)	0(0.073)	0.805(0.035)	0.001(0.035)
**lh_paracentral_thickness**	0.787(0.057)	0(0.057)	0.768(0.073)	0.001(0.073)	0.804(0.036)	0(0.036)

Supplementary Table 6: Regional Contributions to the Person Based Similarity Index (PBSI)						
	**Healthy Individuals**		**Schizophrenia**		**Bipolar Disorder**	
**Variable removed in the leave-one-out analyses**	**Recalculated PBSI following removal of the region shown in column 1**	**Regional Contribution to the PBSI Score***	**Recalculated PBSI following removal of the region shown in column 1**	**Regional Contribution to the PBSI Score ***	**Recalculated PBSI following removal of the region shown in column 1**	**Regional Contribution to the PBSI Score ***
**lh_precuneus_thickness**	0.787(0.057)	0(0.057)	0.766(0.072)	0.001(0.072)	0.803(0.036)	0.001(0.036)
**Right-Lateral-Ventricle**	0.937(0.023)	0.029(0.023)	0.938(0.017)	0.025(0.017)	0.939(0.016)	0.024(0.016)
**Left-Lateral-Ventricle**	0.925(0.022)	0.018(0.022)	0.935(0.018)	0.022(0.018)	0.937(0.017)	0.021(0.017)
**Left-Accumbens-area**	0.89(0.038)	0.017(0.038)	0.895(0.032)	0.017(0.032)	0.9(0.03)	0.016(0.03)
**Right-Accumbens-area**	0.892(0.036)	0.015(0.036)	0.895(0.032)	0.017(0.032)	0.899(0.03)	0.017(0.03)
**Right-Amygdala**	0.897(0.04)	0.011(0.04)	0.901(0.031)	0.011(0.031)	0.907(0.03)	0.008(0.03)
**Left-Pallidum**	0.897(0.039)	0.011(0.039)	0.901(0.03)	0.012(0.03)	0.906(0.03)	0.01(0.03)
**Right-Pallidum**	0.897(0.039)	0.01(0.039)	0.902(0.029)	0.01(0.029)	0.907(0.03)	0.009(0.03)
**Left-Amygdala**	0.899(0.037)	0.008(0.037)	0.9(0.032)	0.012(0.032)	0.907(0.03)	0.008(0.03)
**Left-Thalamus**	0.9(0.035)	0.007(0.035)	0.906(0.029)	0.007(0.029)	0.91(0.026)	0.006(0.026)
**Right-Thalamus**	0.902(0.036)	0.006(0.036)	0.907(0.029)	0.005(0.029)	0.91(0.027)	0.006(0.027)
**Left-Hippocampus**	0.913(0.028)	0.005(0.028)	0.918(0.024)	0.006(0.024)	0.921(0.022)	0.005(0.022)
**Left-Putamen**	0.912(0.031)	0.004(0.031)	0.913(0.027)	0.001(0.027)	0.917(0.025)	0.001(0.025)
**Right-Hippocampus**	0.912(0.027)	0.004(0.027)	0.919(0.023)	0.007(0.023)	0.92(0.022)	0.004(0.022)
**Right-Putamen**	0.911(0.032)	0.003(0.032)	0.914(0.027)	0.002(0.027)	0.918(0.024)	0.002(0.024)
**Right-Caudate**	0.904(0.029)	0.003(0.029)	0.914(0.024)	0.001(0.024)	0.914(0.022)	0.002(0.022)
**Left-Caudate**	0.904(0.03)	0.003(0.03)	0.914(0.025)	0.001(0.025)	0.915(0.022)	0.001(0.022)

Values shown are the mean (standard deviation) within each diagnostic group of the sample from the Icahn School of Medicine at Mount Sinai; *Absolute difference between the original PBSI (i.e., including all regions) and the recalculated PBSI (i.e., including all minus one region). A high difference reflects a high contribution for the region excluded.

3. Person-Based Similarity Index for Cortical Thickness within Each Lobe in Schizophrenia

We calculated PBSI-CT scores for each lobe to obtain a finer grained view of the spatial effect of diagnosis. PBSI-CT for each lobe was computed using cortical thickness measures from 24 frontal regions, 16 parietal regions, 14 temporal regions and 10 occipital regions. In the discovery ISMMS sample and the COBRE replication sample, the mean PBSI-CT within each lobe was lower in the patients compared to the healthy individuals (Supplementary Table 7 and Supplementary Figure 4).

Supplementary Table 7: Person-based Similarity Index for Cortical Thickness (PBSI-CT) within each lobe in patients with schizophrenia and healthy individuals

**PBSI-CT**	**Patients with Schizophrenia**	**Healthy Individuals**	**Case-Control Difference**
**ISMMS Discovery Sample**			
**Frontal Lobe**	Mean (sd)=0.67 (0.06)<br>Median=0.69	Mean (sd)=0.68 (0.70)<br>Median=0.70	Z=1.27, P=0.20
**Parietal Lobe**	Mean (sd)=0.82 (0,04)<br>Median=0.82	Mean (sd)=0.83 (0.04)<br>Median=0.84	**Z=2.55, P=0.01**
**Temporal Lobe**	Mean (sd)=0.84 (0.06)<br>Median=0.85	Mean (sd)=0.87 (0.04)<br>Median=0.88	**Z=4.36, P=1.3×10^{-5}**
**Occipital Lobe**	Mean (sd)=0.74 (0.09)<br>Median=0.77	Mean (sd)=0.79 (0.07)<br>Median=0.81	**Z=4.76, P=2×10^{-8}**
**COBRE Replication Sample**			
**Frontal Lobe**	Mean (sd)=0.64 (0.06)<br>Median=0.64	Mean (sd) =0.65 (0.06)<br>Median=0.66	Z=1.91, P=0.06
**Parietal Lobe**	Mean (sd)=0.71 (0.09)<br>Median=0.74	Mean=0.77 (0.07)<br>Median=0.78	**Z=4.91, P=4.9×10^{-7}**
**Temporal Lobe**	Mean (sd)=0.77 (0.06)	Mean (sd)=0.78 (0.06)	Z=1.64, P=0.102

**Supplementary Table 7: Person-based Similarity Index for Cortical Thickness (PBSI-CT) within each lobe in patients with schizophrenia and healthy individuals**			
**PBSI-CT**	**Patients with Schizophrenia**	**Healthy Individuals**	**Case-Control Difference**
	Median=0.79	Median=0.80	
**Occipital Lobe**	Mean (sd)=0.92 (0.02)<br>Median=0.93	Mean (sd)=0.93 (0.03)<br>Median=0.94	**Z=2.89, P=0.004**
Statistics are based on Mann-Whitney U tests. Bold font indicates significant findings after applying a Family Discovery Rate correction; ISMMS=Icahn School of Medicine at Mount Sinai; sd=standard deviation.			

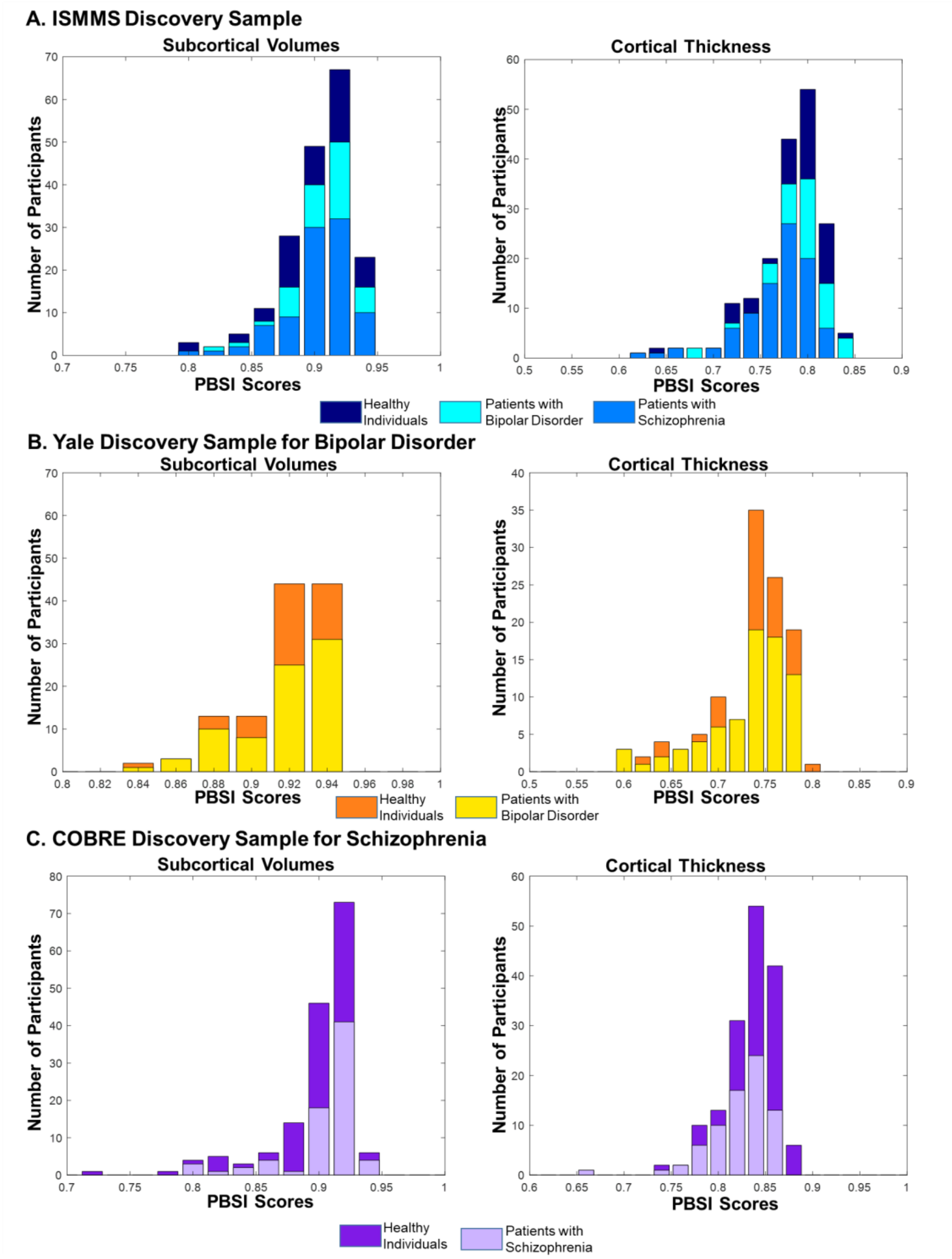

Supplementary Figure 3: Stacked distribution of the PBSI scores in each study sample.

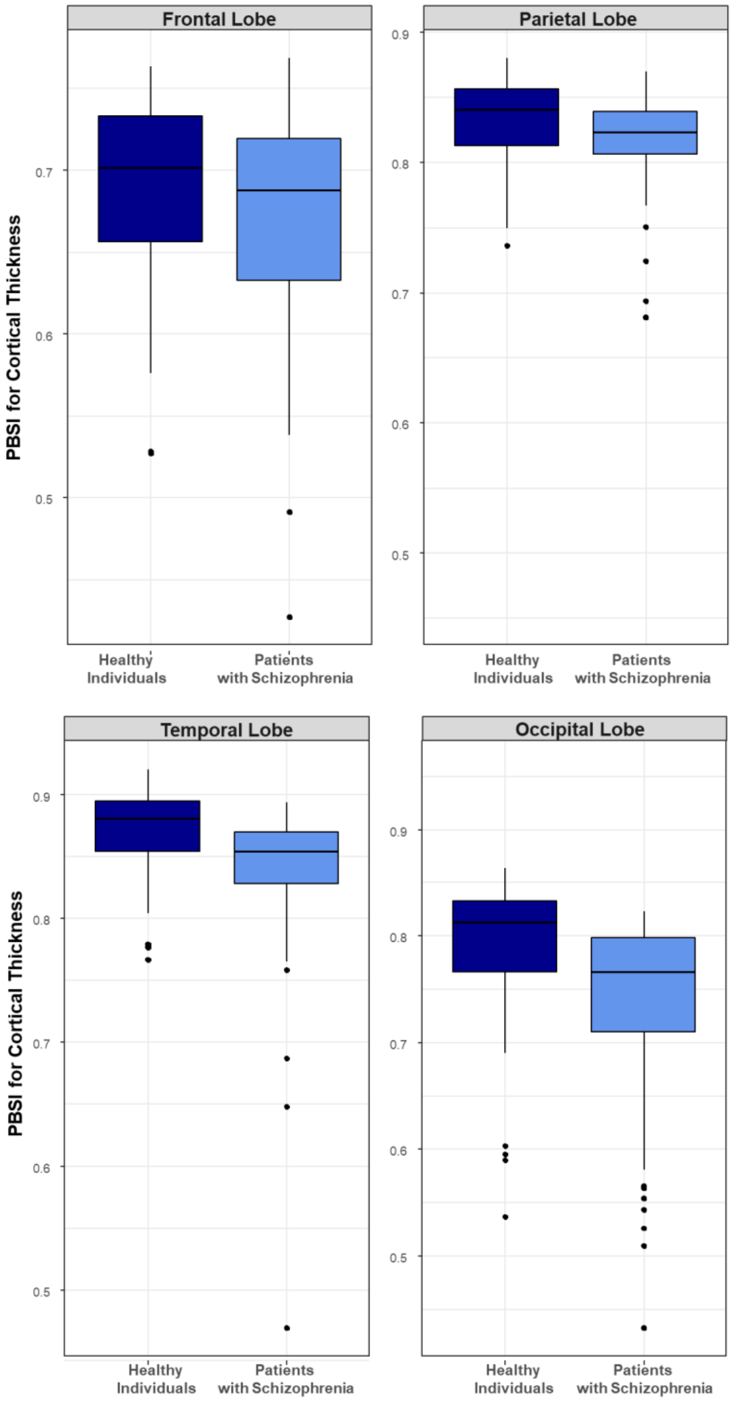

Supplementary Figure 4: Person Based Similarity Index for cortical thickness in each of the four lobes

Patients with schizophrenia had lower scores in all lobes than healthy individuals (Table S7). The results shown are from the ISMMS discovery sample; the same pattern was noted for the COBRE replication samples**.**

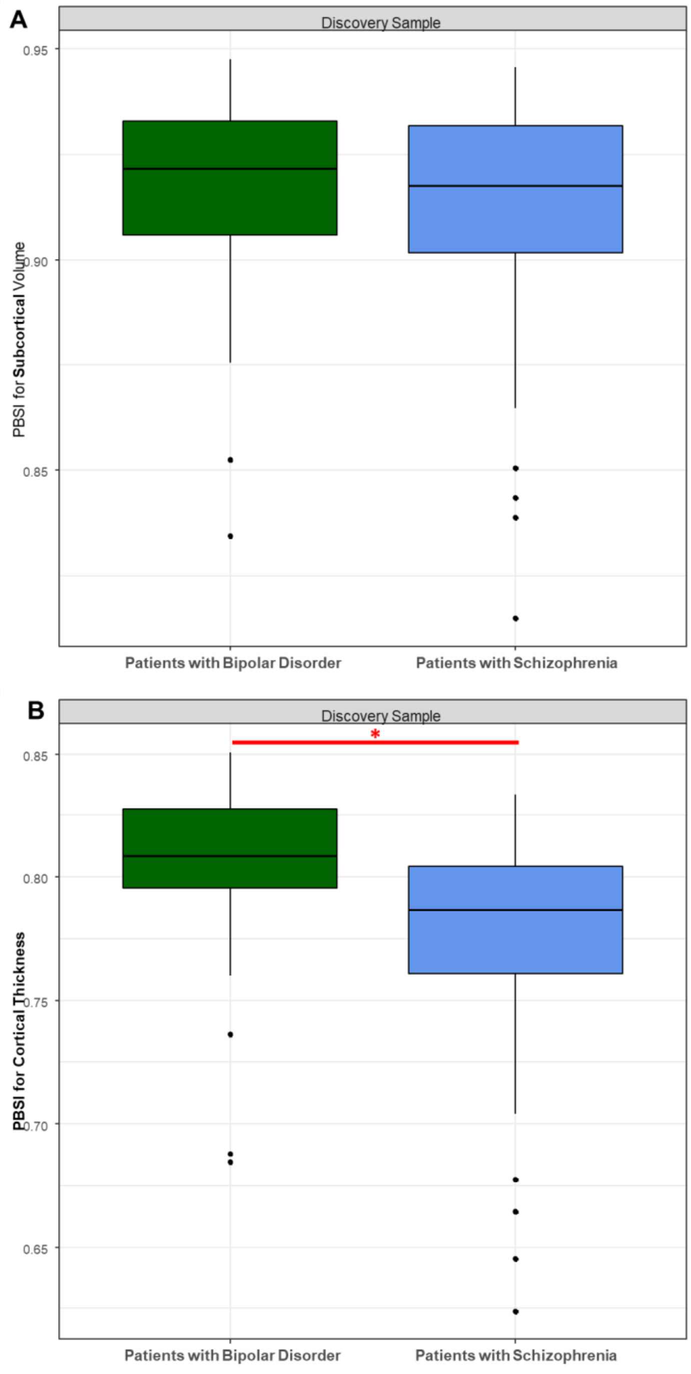

Supplementary Figure 5: Person Based Similarity Index for subcortical volume (A) and cortical thickness (B) between patients with bipolar disorder and schizophrenia, in the Discovery ISMMS Sample. *: significant difference between patients with bipolar disorder and patients with schizophrenia (p_{FDR}<0.05)

4. Recomputation of the PBSI Scores after combining both psychiatric groups
In the ISMMS Group, we recalculated the PBSI Scores (SV and CT, separately) for each patient, after combining both diagnostic groups (bipolar and schizophrenia). For the PBSI-SV, we did not find any significant group differences (Kruskal-Wallis Test, p=0.8). For the PBSI-CT, we found a significant group difference (Kruskal-Wallis Test, p=0.002, K=12.2), with both patient groups showing lower scores than the healthy individuals (BD vs healthy: T=2.4, p=0.048; SZ vs healthy: T=3.45, p=0.002) (Supplementary Figure S6).

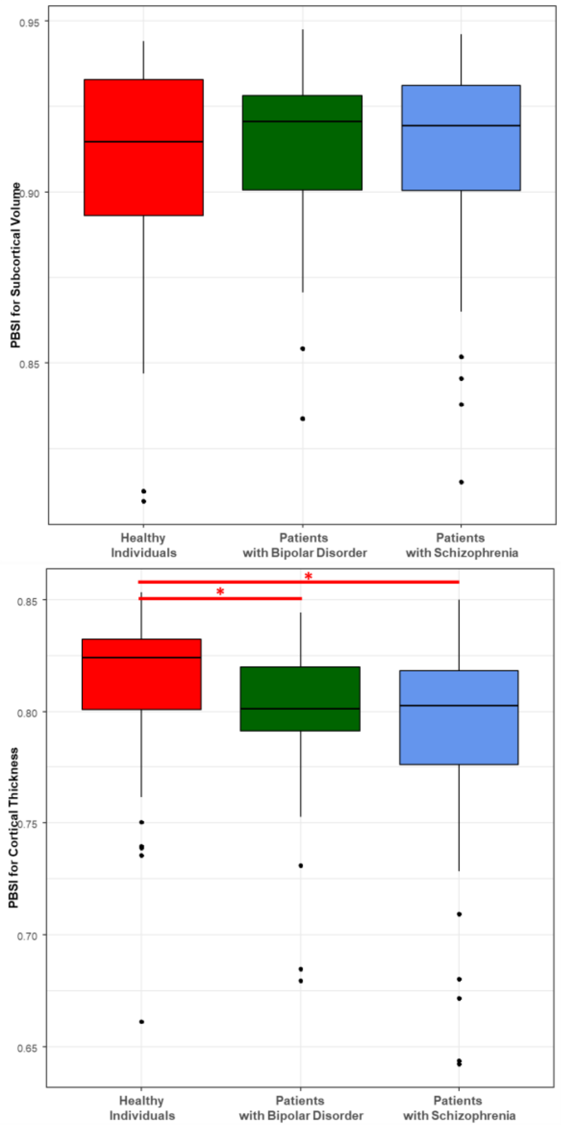

Supplementary Figure 6: Person Based Similarity Index for subcortical volume (A) and cortical thickness (B) between all patients and healthy individuals, in the Discovery ISMMS Sample. *: significant difference a p<0.05 adjusted for multiple comparisons.

References

1. Association AP. Diagnostic and statistical manual of mental disorders. American Psychiatric Publishing, 2013.
2. First MB, Williams JBW, Karg RS, Spitzer RL. Structured clinical interview for DSM-5, research version2015.
3. Wechsler D. Wechsler Abbreviated Scale of Intelligence-2nd edition. NCS Pearson, 2011.
4. Gardner DM, Murphy AL, O'Donnell H, Centorrino F, Baldessarini RJ. International consensus study of antipsychotic dosing. The American journal of psychiatry. 2010; 167(6): 686-93.
5. Aine CJ, Bockholt HJ, Bustillo JR, Canive JM, Caprihan A, Gasparovic C, et al. Multimodal Neuroimaging in Schizophrenia: Description and Dissemination. Neuroinformatics. 2017; 15(4): 343-64.
6. First MB, Spitzer RL, Gibbon M, Williams JBW. Structured clinical interview for DSM-IV axis I diorders-research version. Biometric Research, 1997.
7. Kay SR, Fiszbein A, Opler LA. The positive and negative syndrome scale (PANSS) for schizophrenia. Schizophrenia bulletin. 1987; 13(2): 261-76.
8. Segonne F, Dale AM, Busa E, Glessner M, Salat D, Hahn HK, et al. A hybrid approach to the skull stripping problem in MRI. NeuroImage. 2004; 22(3): 1060-75.
9. Fischl B, Salat DH, Busa E, Albert M, Dieterich M, Haselgrove C, et al. Whole brain segmentation: automated labeling of neuroanatomical structures in the human brain. Neuron. 2002; 33(3): 341-55.
10. Fischl B, Salat DH, van der Kouwe AJ, Makris N, Segonne F, Quinn BT, et al. Sequence-independent segmentation of magnetic resonance images. NeuroImage. 2004; 23 Suppl 1: S69-84.
11. Sled JG, Zijdenbos AP, Evans AC. A nonparametric method for automatic correction of intensity nonuniformity in MRI data. IEEE Trans Med Imaging. 1998; 17(1): 87-97.
12. Fischl B, Liu A, Dale AM. Automated manifold surgery: constructing geometrically accurate and topologically correct models of the human cerebral cortex. IEEE Trans Med Imaging. 2001; 20(1): 70-80.
13. Segonne F, Pacheco J, Fischl B. Geometrically accurate topology-correction of cortical surfaces using nonseparating loops. IEEE Trans Med Imaging. 2007; 26(4): 518-29.
14. Pintzka CW, Hansen TI, Evensmoen HR, Haberg AK. Marked effects of intracranial volume correction methods on sex differences in neuroanatomical structures: a HUNT MRI study. Front Neurosci. 2015; 9: 238.
