# Supplementary material for: Personalized estimates of morphometric similarity in bipolar disorder and schizophrenia
Source: NPJ Schizophr. 2020 Dec 4;6:39. doi: 10.1038/s41537-020-00128-x (PMC7718905; doi:10.1038/s41537-020-00128-x)
Supplement: Supplementary file 2 — Reporting Summary [file 41537_2020_128_MOESM2_ESM.pdf]

nature research

Corresponding author(s): Sophia Frangou

Last updated by author(s): Jul 15, 2020

Reporting Summary

Nature Research wishes to improve the reproducibility of the work that we publish. This form provides structure for consistency and transparency in reporting. For further information on Nature Research policies, see our Editorial Policies and the Editorial Policy Checklist.

Statistics

For all statistical analyses, confirm that the following items are present in the figure legend, table legend, main text, or Methods section.

n/a	Confirmed	
☐	☒	The exact sample size (*n*) for each experimental group/condition, given as a discrete number and unit of measurement
☐	☒	A statement on whether measurements were taken from distinct samples or whether the same sample was measured repeatedly
☐	☒	The statistical test(s) used AND whether they are one- or two-sided<br>*Only common tests should be described solely by name; describe more complex techniques in the Methods section.*
☐	☒	A description of all covariates tested
☐	☒	A description of any assumptions or corrections, such as tests of normality and adjustment for multiple comparisons
☐	☒	A full description of the statistical parameters including central tendency (e.g. means) or other basic estimates (e.g. regression coefficient) AND variation (e.g. standard deviation) or associated estimates of uncertainty (e.g. confidence intervals)
☐	☒	For null hypothesis testing, the test statistic (e.g. *F*, *t*, *r*) with confidence intervals, effect sizes, degrees of freedom and *P* value noted<br>*Give P values as exact values whenever suitable.*
☒	☐	For Bayesian analysis, information on the choice of priors and Markov chain Monte Carlo settings
☒	☐	For hierarchical and complex designs, identification of the appropriate level for tests and full reporting of outcomes
☐	☒	Estimates of effect sizes (e.g. Cohen's *d*, Pearson's *r*), indicating how they were calculated

Our web collection on statistics for biologists contains articles on many of the points above.

Software and code

Policy information about availability of computer code

Data collection	No software was used
Data analysis	Freesurfer (publicly available) and the MATLAB function used to compute the PBSI score is available at: https://www.mathworks.com/matlabcentral/fileexchange/69158-similarityscore.

For manuscripts utilizing custom algorithms or software that are central to the research but not yet described in published literature, software must be made available to editors and reviewers. We strongly encourage code deposition in a community repository (e.g. GitHub). See the Nature Research guidelines for submitting code & software for further information.

Data

Policy information about availability of data

All manuscripts must include a data availability statement. This statement should provide the following information, where applicable:

- Accession codes, unique identifiers, or web links for publicly available datasets
- A list of figures that have associated raw data
- A description of any restrictions on data availability

The data that support the findings of this study are available from the corresponding author upon reasonable request.

Field-specific reporting

Behavioural & social sciences study design

All studies must disclose on these points even when the disclosure is negative.

Study description	Quantitative comparison between patients with schizophrenia, patients with bipolar disorder and healthy individuals in terms of their brain structural morphometry
Research sample	The research involved 3 independent samples: patients with schizophrenia (n=93) or bipolar disorder (n=44) and healthy individuals (n=52) acquired at the Icahn School of Medicine at Mount Sinai. Independently acquired data on schizophrenia (COBRE sample: patients=75; health individuals=87) provided by the Center of Biomedical Research Excellence (COBRE) (http://coins.trendscenter.org); and bipolar disorder (Yale sample: patients=78; healthy individuals=41) acquired at the Olin Neuropsychiatric Research Center, Yale University, Hartford, CT, USA.<br>Healthy individuals were recruited by advertisement in the local press.
Sampling strategy	Three independent samples were used to test the replicability of findings across samples and show that the results were independent of acquisition protocol and site.<br>ISMMS and Yale Samples: Regardless of diagnosis, eligible participants had an intelligence quotient >70 as assessed with the Wechsler Abbreviated Scale of Intelligence (WASI-II), had no lifetime history of DSM-5 substance use disorder or of any medical or neurological disorder. For all participants, assessments of diagnosis and rating of psychopathology were undertaken by an experienced clinician using the Structured Clinical Interview for DSM-5 and the 24-item Brief Psychiatric Rating Scale (BPRS).<br>COBRE Sample: The diagnostic status of the COBRE participants was ascertained according to the Diagnostic and Statistical Manual of Mental Disorders, Fourth Edition (DSM-IV) using the Structured Clinical Interview using the Structured Clinical Interview for DSM-IV Axis I Disorders. Healthy individuals did not have any personal or family (up to second degree relatives) of psychiatric disorders. All participants were screened to exclude those with a history of neurological disorder, mental retardation, severe head trauma, substance abuse or dependence within the last 12 months and MRI contra-indications. Psychopathology in patients assessed with the Positive and Negative Syndrome Scale (PANSS). The COBRE Stability Clinic determined retrospective stability from relevant psychiatric records documenting that no change in symptomatology or type/dose of psychotropic medications occurred during the three months prior to the referral.
Data collection	Pen and Paper instruments for IQ assessment and for clinical assessments. All instruments are available upon request. Brain imaging data collection was implemented using hardware and software available in each 3T MR scanner (within each site).
Timing	ISMMS Sample: from 07/20/2014 to 05/30/2018<br>Yale Sample: from 04/20/2009 to 01/20/2014<br>COBRE Sample: This sample is an open-access collection of neuroimaging data in schizophrenia (http://cobre.mrn.org; http://coins.mrn.org).
Data exclusions	No data were excluded.
Non-participation	No participants dropped out
Randomization	Participants were either patients fulfilling diagnostic criteria for schizophrenia, bipolar disorder, or healthy participants; the groups were demographically matched.

Reporting for specific materials, systems and methods

We require information from authors about some types of materials, experimental systems and methods used in many studies. Here, indicate whether each material, system or method listed is relevant to your study. If you are not sure if a list item applies to your research, read the appropriate section before selecting a response.

Materials & experimental systems

n/a	Involved in the study	
☒	☐	Antibodies
☒	☐	Eukaryotic cell lines
☒	☐	Palaeontology and archaeology
☒	☐	Animals and other organisms
☐	☒	Human research participants
☐	☒	Clinical data
☒	☐	Dual use research of concern

Methods

n/a	Involved in the study	
☒	☐	ChIP-seq
☒	☐	Flow cytometry
☐	☒	MRI-based neuroimaging

Human research participants

Policy information about studies involving human research participants

Population characteristics	See above
Recruitment	Participants were recruited on the basis of specific characteristics as described above.
Ethics oversight	Institutional Review Board of the Icahn School of Medicine at Mount Sinai; Institutional Review Board at Hartford Hospital and Yale University; Institutional Review Board of the University of New Mexico

Note that full information on the approval of the study protocol must also be provided in the manuscript.

Clinical data

Policy information about clinical studies

All manuscripts should comply with the ICMJEguidelines for publication of clinical research and a completedCONSORT checklist must be included with all submissions.

Clinical trial registration	This study is not a clinical trial
Study protocol	This study is not a clinical trial
Data collection	This study is not a clinical trial
Outcomes	This study is not a clinical trial

Magnetic resonance imaging

Experimental design

Design type	Structural MRI only
Design specifications	n/a
Behavioral performance measures	n/a

Acquisition

Imaging type(s)	structural magnetic resonance imaging data
Field strength	3 Tesla
Sequence & imaging parameters	ISMMS Sample: a T1-weighted, 3D magnetization-prepared rapid gradient-echo (MPRAGE) sequence with the following parameters: Repetition time (TR)=2400ms; Time to Echo (TE)=2.07ms and Inversion time (TI)=1000ms, voxel size=0.8mm isotropic, flip angle=8°, field of view (FOV)=256×256×179mm3, matrix size=320×320, bandwidth=240 Hz/Pixel, echo spacing=7.6ms, in-plane acceleration GRAPPA (GeneRalized Autocalibrating Partial Parallel Acquisition) factor 2.<br>Yale Sample: T1-weighted, 3D MPRAGE sequence with the following parameters: TR/TE/TI= 2200/4.13/766ms, voxel size=0.8mm isotropic, flip angle=13°, image size=240×320×208 voxels.<br>COBRE Sample: a multi-echo (number of echos=5) MPRAGE (MEMPR) sequence with the following parameters: TR=2530ms; five TE=1.64, 3.5, 5.36, 7.22, 9.08ms, TI=900ms, 1mm isotropic resolution. FOV=256x256, flip angle=7°, matrix size=256x256x176, pixel bandwidth=650 Hz.
Area of acquisition	Whole Brain

Diffusion MRI ☐ Used ☒ Not used

Preprocessing

Preprocessing software	FreeSurfer 5.3.0
Normalization	Automated Talairach transformation, segmentation of the subcortical white matter and deep gray matter volumetric structures, intensity normalization, tessellation of the boundary between the gray and white matter, automated topology correction
Normalization template	Talairach
Noise and artifact removal	removal of non-brain tissue using a hybrid watershed/surface deformation procedure
Volume censoring	n/a

Statistical modeling & inference

Model type and settings	Within each site, between-group differences were tested using Mann-Whitney U tests. Correlation analyses were computed using Spearman's correlation analyses.
Effect(s) tested	Case-control differences between diagnostic groups. We also assessed the association of PBSI scores with age, sex, and cortical thickness or subcortical volume measures in all participants and, in patients with symptom ratings, and medication status.

Specify type of analysis: ☒ Whole brain ☐ ROI-based ☐ Both

Statistic type for inference (See Eklund et al. 2016)	n/a
Correction	FDR

Models & analysis

n/a	Involved in the study
☒	☐ Functional and/or effective connectivity
☒	☐ Graph analysis
☒	☐ Multivariate modeling or predictive analysis
